# A method for PCR-free library preparation for sequencing palaeogenomes

**DOI:** 10.1371/journal.pone.0319573

**Published:** 2025-03-19

**Authors:** Kirstin Henneberger, Axel Barlow, Federica Alberti, Michaela Preick, Silviu Constantin, Doris Döppes, Wilfried Rosendahl, Michael Hofreiter, Johanna L. A. Paijmans

**Affiliations:** 1 Institute for Biochemistry and Biology, University of Potsdam, Potsdam, Germany; 2 School of Environmental and Natural Sciences, Bangor University, Bangor, United Kingdom; 3 Department of Geospeleology and Paleontology, Emil Racovita Institute of Speleology, Bucharest, Romania; 4 Centro Nacional de Investigacion sobre la Evolucion Humana, CENIEH, Burgos, Spain; 5 Reiss-Engelhorn-Museen, Mannheim, Germany; 6 Curt-Engelhorn-Centre for Archaeometry, Mannheim, Germany; 7 Department of Zoology, University of Cambridge, Cambridge, United Kingdom; University of Warsaw, POLAND

## Abstract

In recent years, methodological advances have substantially improved our ability to recover DNA molecules from ancient samples, raising the possibility to sequence palaeogenomes without PCR amplification. Here we present an amplification-free library preparation method based on a benchmark library preparation protocol in palaeogenomics based on single-stranded DNA, and demonstrate suitability of the new method for a range of sample types. Furthermore, we use the method to generate the first amplification-free nuclear genome of a Pleistocene cave bear, and analyse the resulting data in the context of cave bear population genetics and phylogenetics using standard genomic clustering analyses. We find that the PCR-free adaptation provides endogenous DNA contents, GC contents and fragment lengths consistent with the standard protocol, although with reduced conversion efficiency, and shows no biases in downstream population clustering analyses. Our amplification-free library preparation method could find application in experimental designs where the original template molecule needs to be characterised more directly.

## Introduction

A long-standing assumption in palaeogenetic research is that the genetic material present in ancient samples occurs in very low quantities, and that the amount of exogenous DNA always far outweighs the target or endogenous DNA. In order to overcome this limitation, genetic analysis pipelines have always included a PCR amplification step in order to generate enough genetic material for sequencing; either by species-specific primers, or library amplification prior to sequencing. For this reason, PCR has often, and with justification, been hailed as the most important development that enabled palaeogenetic research, leading to many unprecedented insights into evolution, ecology, human history and disease [reviewed in e.g. 1].

In recent years, thanks to the development of new DNA extraction methods [e.g. 2], library preparation methods [3–5] and improved tissue sampling strategies [6–8], the standard expectations regarding the prevalence of DNA in an ancient sample have changed. Yields over 100 ng DNA per extract have been reported for high endogenous content Pleistocene samples (S1 Fig in S1 File), which for a typical mammalian species would correspond to over 16,000 diploid copies of the nuclear genome, if all extracted DNA were endogenous. Such DNA yields could make sequencing of even a high coverage genome without PCR amplification achievable. Excluding PCR from the library construction protocol would further avoid duplications as well as artefacts caused by amplification [9–11], with the potential to increase usable data output from the sequencer [12,13]. Furthermore, amplification-free recovery of DNA would allow for a more direct and unbiased characterisation of the genetic material preserved in the ancient sample, potentially leading to a better understanding of e.g. copy number variations, or the relative presence of alleles in a poolseq experimental design.

To date, no specific protocol for recovering ancient DNA without the need for PCR amplification exists. Although amplification-free protocols have been developed for modern DNA [14], the recovery of DNA from ancient and other degraded samples require specialised protocols that target very short and damaged template molecules [3]. The requirements for standard and/or commercial amplification-free protocols (25-200 ng of several hundred bp long double-stranded DNA fragments; see e.g. https://www.illumina.com/products/by-type/sequencing-kits/library-prep-kits/dna-pcr-free-prep.html) are very difficult to achieve for ancient samples. To our knowledge, only one amplification-free protocol has been developed for low input single-stranded DNA, for recovering cell-free fetal DNA [15]. Although this protocol is based on the single-stranded library protocol for ancient DNA, it is unclear how its adaptations would impact the recovery of ancient DNA, and the downstream compatibility of the data with the single-stranded protocol [16–18].

Here, we present an amplification-free adaptation of a well-established protocol for ancient DNA, which was the first single-stranded library preparation developed for Illumina sequencing [3,19]. We show the effectiveness of our amplification-free protocol through application to a range of samples of various ages (from museum samples to Pleistocene sub-fossil samples) and tissue types (bone, preserved skin, formalin fixed tissue), and compare its performance to the standard single-stranded library preparation protocol. Finally, we utilise the technique to sequence a Pleistocene nuclear genome without any PCR amplification prior to sequencing, and compare the data with those recovered using the standard single-stranded protocol to gauge for downstream biases. Our amplification-free protocol could have application in fields where low-quantity input DNA is common, such as palaeogenomics, museomics or forensics, and where a reduction of potential PCR bias is desired.

## Materials and methods

### Library protocol

**Fig 1:**
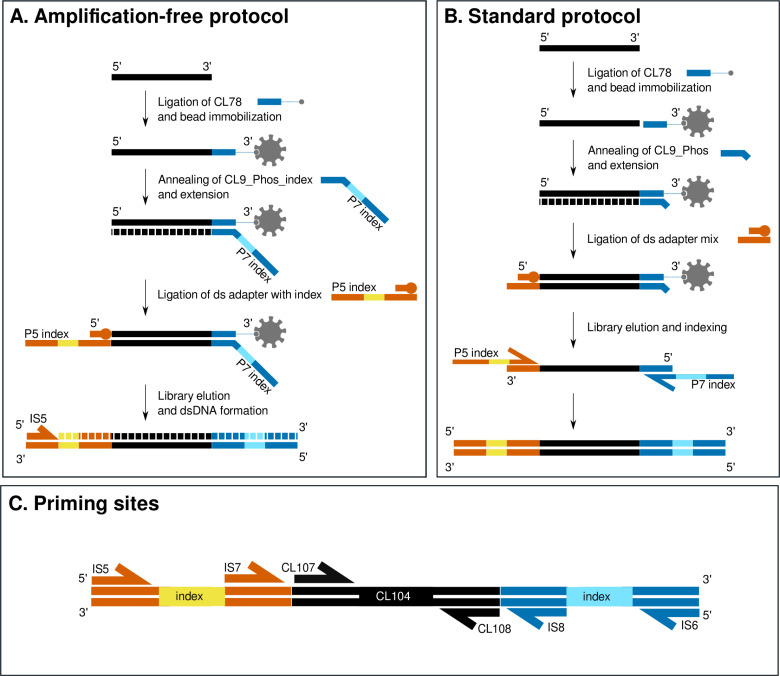
Overview of both the amplification-free protocol (A) and the standard single-stranded protocol **(B)** [3,19]. Denaturation and immobilisation steps are identical between the protocols. The priming sites for the quantification assays are indicated in panel C. For more details, see S1 Protocol and S5 Table in S1 File.

The amplification-free protocol is based on the first published single-stranded library method for ancient DNA (Fig 1) [3,4,19]. After removal of terminal phosphate groups and denaturation of the template DNA, a biotinylated adapter is ligated to the resulting single-stranded template molecules. Then, instead of annealing a truncated adapter to the biotinylated adapter, our new amplification-free protocol anneals a specifically modified oligonucleotide consisting of the full adapter sequence including the inline index, which is then extended along the template. The second adapter is added using blunt-end ligation, again using a modified oligonucleotide mix that contains the full-length double-stranded adapter including the inline barcode. After another heat denaturation to release the new template molecule, it is then converted into a double-stranded molecule using a fill-in reaction in which the original template DNA molecule is displaced. This fill-in reaction replaces the indexing PCR in the standard single-stranded protocol (Fig 1). A detailed protocol is presented as S1 Protocol in S1 File.

### Investigation of library conversion

We investigated the conversion of template molecules into the final sequencing library using two experiments:

#### Experiment 1.

To calculate the conversion rate – defined here as the total number of molecules in the final product versus the number of molecules with library adapters – of the amplification-free protocol compared to the standard single-stranded protocol, both types of libraries were prepared from the test oligo CL104 (Fig 1C; [also see 3]), and the conversion rates calculated using qPCR as described in Gansauge and Meyer [3]. The procedure in brief: 1 µl of a 0.1 µ M dilution of the CL104 oligo was used as input for library preparation, using a total of five replicates each (one replicate set of the original six failed). Following library preparation, two qPCR assays and a standard dilution series were used to determine the number of molecules successfully converted into library molecules, as described in Gansauge and Meyer [3], using triplicate qPCR reactions for each library. qPCR assay A involved amplification using the IS7/IS8 primer pair, which amplifies only complete library molecules, indicating successful ligation of a P5 and P7 adapter on each end of the test oligo CL104 (Fig 1C). Assay B involved amplification using primers CL107/CL108, which detect oligo CL104 regardless of whether it has been successfully converted into a library molecule or not (Fig 1C). A standard dilution series of a known quantity of CL104 (amplified from pUC19 DNA) was used for absolute quantification. qPCR analysis was carried out on a Roche LightCycler 480 and intensity values converted into Ct values using R v4.1.0 [20] with the package ‘qpcR’ [21].

#### Experiment 2.

To further compare library conversion of the amplification-free and standard protocol when applied to real samples, an assay of six ancient bones samples (three replicates each; Table 1) was used. 50 mg of bone powder was taken from each sample and DNA extracted following Dabney et al. [2]. These extracts were then used to prepare both amplification-free and standard (i.e. PCR amplified) single-stranded libraries. By using qPCR assay A (described above), we performed absolute quantification of the successfully converted library molecules.

**Table 1:** Details of samples used in this study.

Sample name	Organism	Sample type	Approximate age	Experiment	Sample provider
NK64	steppe bison	petrosa dex	39.0 ka BP uncal	2	Reiss-Engelhorn-Museen
NK65	steppe bison	petrosa sin	40.1 ka BP uncal	2	Reiss-Engelhorn-Museen
NK67	steppe bison	petrosa sin	41.4 ka BP uncal	2	Reiss-Engelhorn-Museen
NK69	steppe bison	petrosa	39.4 ka BP uncal	2	Reiss-Engelhorn-Museen
NK71	aurochs	petrosa sin	42.6 ka BP uncal	2	Reiss-Engelhorn-Museen
MENG21	aurochs	petrosa sin	43.6 ka BP uncal	2	Reiss-Engelhorn-Museen
33961	king cobra	formalin-fixed, ethanol preserved	1970s AD	3	Natural History Museum Berlin
PLI-19	linsang	soft tissue	1896 AD	3	Naturalis Museum Leiden
50746NB	leopard	nasal bone	1962 AD	3	Natural History Museum Berlin
70423NB	leopard	nasal bone	unknown (historical)	3	Natural History Museum Berlin
56097ST	leopard	soft tissue	1904 AD	3	Natural History Museum Berlin
56407ST	leopard	soft tissue	1907 AD	3	Natural History Museum Berlin
SP347	cave bear	bone - not compact	Pleistocene	3	Institute of Palaeontology, University of Vienna
PM-V6-IV-II	cave bear	petrous	Pleistocene	3	Emil Racovita Institute of Speleology, Romania
PM-T6-I-II-102	cave bear	petrous	Pleistocene	3	Emil Racovita Institute of Speleology, Romania
PU-D2-VIII-101	cave bear	petrous	Pleistocene	3	Emil Racovita Institute of Speleology, Romania
PM-T6-I-II-101	cave bear	bone - compact	Pleistocene	3	Emil Racovita Institute of Speleology, Romania
O34-16	bear	petrous	Pleistocene	3, 4	Emil Racovita Institute of Speleology, Romania

### Data consistency and performance on different sample types

In order to test the efficacy of the amplification-free protocol for different tissue types and preservation contexts, a range of samples were selected for sequencing using both the amplification-free and the standard single-stranded library preparation protocols.

#### Experiment 3.

DNA was extracted from Pleistocene bear bone material (compact and non-compact), archival leopard bone, formalin-fixed snake tissue in ethanol, and preserved skin samples from leopards and an Asiatic linsang (Table 1). Bone samples were extracted following Dabney et al. [2]. For the tissue samples, digestion was performed using a non-destructive buffer containing guanidine-thiocyanate [22], followed by the washing and binding steps from Dabney et al. (2013) [2,23,24]. Between 7 and 13 ng of DNA extract were used for the preparation of both the single-stranded libraries and the amplification-free libraries, as input amounts over 13 ng negatively impacts the efficiency of the single-stranded protocol [3] (S1 Table in S1 File). Libraries were then sequenced on the NextSeq 500 platform using custom primers CL72 and Gesaffelstein [3,25] generating 75 bp single-end reads (S2 Table in S1 File).

Adapter sequences were trimmed from the raw reads using cutadapt v4 [26], using a minimum length filter of 30 bp and a minimal match between read and adapter sequence of 1 bp (-O 1), and then mapped to an appropriate reference genome available at the time of data processing (Tables 1 and S2 in S1 File) using the ‘aln’ algorithm of bwa [27] with the program’s default parameters (-n 0.04). SAMtools v1.12 [28] was used to discard reads with low mapping quality (MapQ < 30). MarkDupsByStartEnd v0.2.1 (https://github.com/dariober/Java-cafe) was used to remove duplicates that share the same 5’-end and 3’-end positions. AMBER (https://github.com/tvandervalk/AMBER; commit b8fa6fe) [29] was used for generating terminal C > T deamination, as well as nucleotide misincorporation and read length plots (S2 and S3 Figs in S1 File).

The following additional variables were recorded from the data: endogenous content (calculated as a percentage of filtered reads that map to the reference genome), duplication rate (calculated as the percentage of reads flagged as duplicates from the total mapped reads), recovered fragment lengths, and GC content. All these statistics were extracted directly from the bam files using samtools and standard BASH code. Statistical analyses and plotting were performed in R v4.1.0 including utilities from the *tidyverse* library [30].

### Amplification free Pleistocene genome

A Late Pleistocene cave bear from Peștera cu Oase cave in Romania (“O34-16”) was selected for whole genome sequencing to 1x coverage, to validate the consistency of data generated from the amplification-free library for population genetic analyses.

#### Experiment 4.

75 bp single-end sequencing was carried out on the amplification-free library of sample O34-16, aiming for 1x coverage. Further sequencing (10 million reads) was additionally carried out for the standard single-stranded library from the same specimen to assess library complexity, and this sequencing run employed 75 bp paired-end sequencing (S2 Table in S1 File). Reads were trimmed as described above (using cutadapt and discarding reads less than 30bp), and overlapping paired-end reads were merged using Flash v1.2.11 [31] with a minimum overlap (-m) of 10bp and a maximum (-M) of 76bp. All data were then mapped as described above and analysed alongside previously published cave, brown and polar bear genomic data [32,33] (S4 Table in S1 File).

Principal component analysis was carried out by calculating an allele covariance matrix in ANGSD v0.933 [34] by sampling a random base for each position (doIBS 1), ignoring reads with a base or mapping quality below 30. Singleton sites, transition sites, and sites where any individual had missing data were also excluded. Principal components were then calculated from the covariance matrix using the eigen() function in R v4.3.1. A neighbour-joining tree was computed by calculating a distance matrix in ANGSD, using the same filters as described above, from which a neighbour-joining tree was generated using the ‘ape’ [35] and ‘phytools’ [36] packages in R. The tree was rooted on a known root node in cave bear phylogenetic relationships, separating the Caucasus and European/Uralian cave bear clades [33].

## Results and discussion

We successfully developed a modified version of the well-established single-stranded library preparation method that avoids the need for a PCR amplification step (S1 Protocol). Using this modified protocol, we were able to successfully generate libraries for all samples investigated in this study (Tables 1 and S1 in S1 File). Using low level sequencing of these libraries (<1 million sequence reads each; S2 Table in S1 File), we were able to assess the performance of the amplification-free library preparation protocol in terms of basic data characteristics such as endogenous content, GC content, duplication and read length. Furthermore, we generated a palaeogenome dataset of a Pleistocene sample without any pre-sequencing amplification (S2 Table in S1 File).

### Conversion

**Fig 2:**
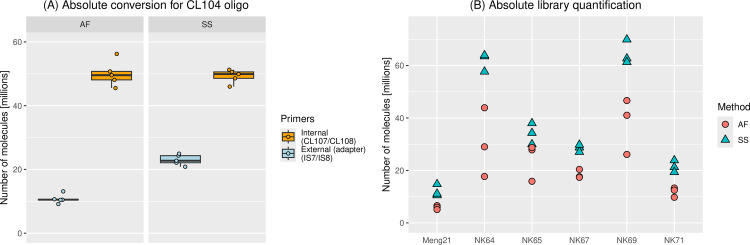
Absolute quantification of libraries. **(A)** Results of experiment 1: Boxplots showing absolute conversion – defined as the total number of molecules in the qPCR input product versus the number of molecules with library adapters – for the amplification-free (AF) and single-stranded (SS) libraries, using the test oligo CL104. The number of CL104 molecules amplifiable with the primers CL107/CL108 (internal; displayed in orange) correspond to the total number of input CL104 molecules (i.e. all those present after library preparation), while the primer pair IS7/IS8 (external; light blue) only amplifies correctly formed library molecules. Points show values for individual experimental replicates. **(B)** Results of experiment 2: Absolute quantification of successfully converted library molecules for six Pleistocene bovid sample extracts, for amplification-free (AF; red circles) and standard single-stranded (SS; blue triangles) libraries.

Comparisons of absolute conversion using the test oligo CL104 indicated that conversion rates for the amplification-free protocol are lower (22%; Fig 2A and S1 Data in S1 File) than for the standard single-stranded protocol (47%). Comparisons using Pleistocene steppe bison bone samples showed the same pattern overall, but revealed that absolute library yield – defined as the absolute quantity of library molecules – for both protocols are in general highly variable between individual samples, and in some cases between experimental replicates (Fig 2B). As expected, we find that library yield is higher for samples where a higher amount of input DNA was used, both for the standard single-stranded protocol and amplification-free method (S1 Table in S1 File and Fig 2B). On average, we achieved 1.8x (range 1.4-2.1x) better yield for the Pleistocene samples with the standard single-stranded library protocol than the amplification-free method, which is in line with the absolute conversion rates measured using the CL104 oligo.

### Data consistency and performance on different sample types

**Fig 3:**
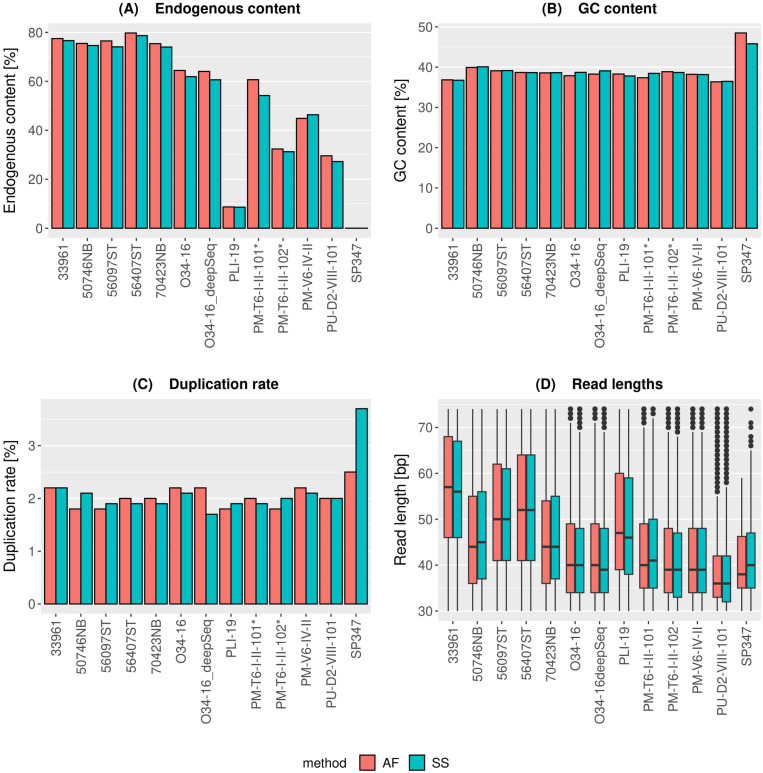
Results of experiment 3: **(A)** Endogenous content, measured by the percentage of trimmed, unique reads that map to the reference genome. **(B)** GC content of mapped reads. **(C)** Percentage of duplications. **(D)** Boxplots showing fragment length distributions of mapped reads in the 30–75 bp range.

Analysis of sequencing data from a wide range of sample types revealed that all investigated variables -- endogenous content, GC content, duplication rates, fragment lengths and terminal deamination -- are highly similar between the amplification-free and standard single-stranded protocols (Figs 3 and S2 in S1 File), which are based on equal amounts of input DNA for each pair of library protocols. Only minor differences between the protocols are observed for one Pleistocene bone sample (SP347) with a very low number of reads mapping to the reference genome (0.02%), suggesting that this is likely a stochastic effect (S2 Table in S1 File). We furthermore calculated all variables also for equal subsamples between the pairs of amplification-free and standard single-stranded libraries (S3 Table in S1 File). The results are consistent between the total and the subsampled data, except for the duplication levels which is lower as expected for subsampled data. Overall, our results indicate that, at the level of coverage tested here, the amplification-free protocol is consistent with the well-established protocol, and can likely be broadly applied across many different sample types. Previous studies have shown that data produced by different library protocols can be highly heterogeneous, with the potential to mislead certain types of analysis [18]. Therefore, the observed consistency in data recovery between amplification-free and standard single-stranded protocol is an important indicator that combining data from these protocols will be less prone to such confounding effects.

### Sequence duplication

A surprising aspect of our sequence data analysis are the similar levels of apparent PCR duplication between amplification-free and standard single-stranded libraries (both approximately 2%; Fig 3 and S2 Table in S1 File). As no PCR amplification was performed in the library preparation, the presence of apparent PCR duplicates in the amplification-free datasets is artefactual. This result does indicate that for the DNA extracts and sequencing depths investigated in this study, the standard single-stranded protocol produces libraries of sufficient complexity that the duplication generated by PCR does not impact the sequencing yield. However, PCR duplication has led to a substantial loss in usable sequence data in other ancient DNA studies; for example a recent ancient giant panda genome dataset [37] contained up to 60% duplicates. In such cases, the amplification-free library preparation could be a potential avenue to improve sequence yield by reducing the amount of data lost to PCR duplication, and thus leverage the budgeted sequencing funds.

**Fig 4:**
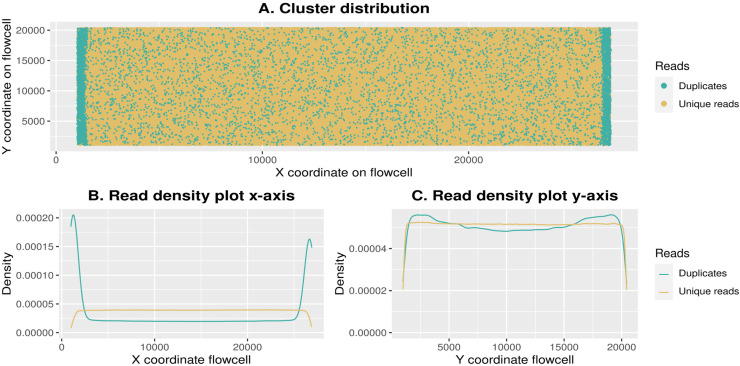
Tile-edge duplications. **(A)** Visual representation of the distribution of unique vs non-unique (duplicate) clusters on the flowcell for sample “O34-16”, amplification-free library (test sequencing). (**B-C)** Density plots for unique and duplicate clusters along the X axis (B) and Y axis (C) for the same library further showing the increased density of duplicates around the edges of the flow cell (deep sequencing).

The absence of PCR duplicates in the amplification free data also provides an opportunity to investigate the potential origin of the artefactual duplication, particularly duplication generated during the sequencing process. We find a spatial pattern of increased duplication at the edges of the flow cell (Fig 4), where duplicates are generated during the sequencing process itself, when the same cluster is falsely identified as different clusters during the imaging stage of sequencing (discussed on https://www.biostars.org/p/229842/). At the levels of sequencing generated in this study, these false optical edge duplications account for approximately half of all duplicates – however it is unknown how this effect scales with increased sequencing levels. In cases where this type of duplications impact data recovery, a coordinate-aware deduplication strategy -- where duplicates are removed for reads near the edge of the flow cell, but not for those in the centre -- could be a method to avoid losing reads to false duplicates.

### Amplification-free Pleistocene genome

**Fig 5:**
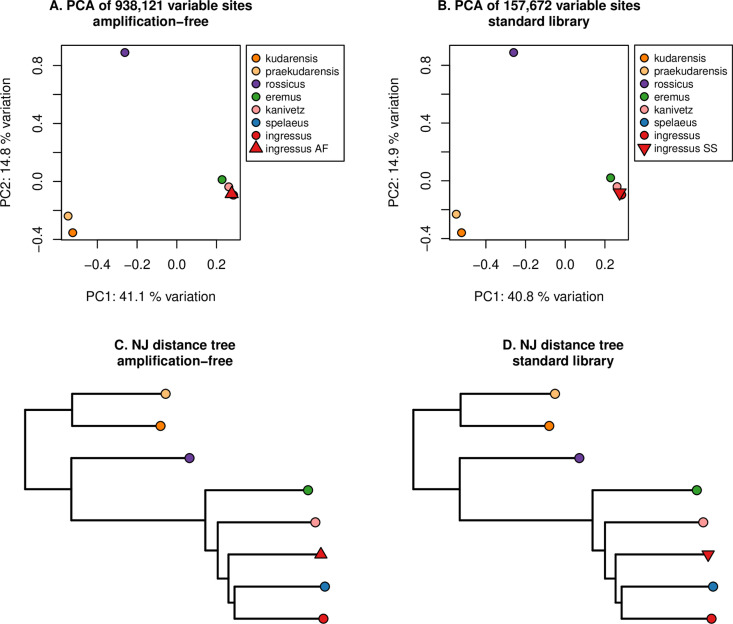
Results of experiment 4: Population clustering of the cave bear data generated with the amplification-free method as well as the standard single-stranded method within existing bear data. (A/B). Principal component analysis of cave bears using the amplification-free (A) and standard (B) methods. (C/D): neighbour-joining phylogenetic analyses using the amplification-free (C) and standard (D) methods.

The consistency of data generated using the amplification-free protocol with that generated with the standard single-stranded library protocol is further confirmed by analysis of the approximately 1x Pleistocene cave bear palaeogenome from Romania (sample “O34-16”). For this sample, we generated 1.5Gb (0.7x) and 0.17Gb (0.07x) of the nuclear genome using the amplification-free and standard single-stranded protocols, respectively. Previous studies have shown that different library preparation protocols can significantly alter the properties of the resulting sequencing data, including fragment length recovery, GC content and damage patterns [17,38], leading to a potential bias in downstream analysis when mixing different library preparation methods in a single study. Furthermore, it has been shown that such biases can impact downstream population clustering analysis, for example resulting in a pronounced branch lengthening effect in phylogenetic trees [29]. In order to test if the amplification-free protocol would result in a bias in downstream analyses, we performed basic population clustering analyses with the datasets generated with the amplification-free protocol and standard single-stranded protocol from the Romanian cave bear. The genetic clustering analyses, both the principal component analysis and the distance-based neighbour-joining tree, generally show no difference when the amplification-free data or the standard single-stranded data is used (Fig 5; based on 938,121 and 157,672 variable sites for the amplification-free and single-stranded data, respectively). For the principal component analysis, the individual clusters as expected with other cave bears from the same evolutionary lineage when considering the first two principal components that together account for more than 50% of the total variation (European cave bears, see [33]; Fig 5). The phylogenetic analyses performed on published data and data generated using the amplification-free protocol shows the same topology for the amplification-free and standard single-stranded libraries, and no apparent differences in branch lengths. We do note that in neither analysis the individual O34-16, identified as an *ingressus* cave bear based on morphology, clusters with the published *ingressus* genome, but rather with the *spelaeus* genome which can be considered a separate evolutionary lineage [39,40]. Further investigations of cave bear population structure is needed to resolve this apparent discrepancy. However, as this result is identical between the two library preparation protocols, this cannot be the result of inadvertent bias of the amplification-free library preparation protocol. Thus, we confirm that the amplification-free protocol enables the sequencing of palaeogenomes without PCR amplification, and that the resulting data can be readily combined and integrated with existing single-stranded datasets for evolutionary and population genetics analyses.

### Limitations of the amplification-free protocol

During the development and testing of the amplification free protocol we noted some limitations. The first is that, unlike the standard single-stranded libraries, the total quantity of library available for sequencing is directly related to the quantity of input DNA. Repeated quantification and sequencing runs will rapidly diminish the available library stock, unlike PCR amplified libraries where a large stock can be amplified for use in many experiments. This means that the level of coverage that can be achieved for an individual library is limited by its volume, rather than complexity. To achieve the 1x coverage (2.9 mapped Gb) for sample O34-16, 3.17µl of the final library product (~25µl) was used for sequencing, meaning that this particular library would be limited to an estimated 4x sequence coverage. Although subsequent amplification of the amplification-free library is possible using standard primers, this would remove the unique aspect of the approach used to generate it.

**Fig 6:**
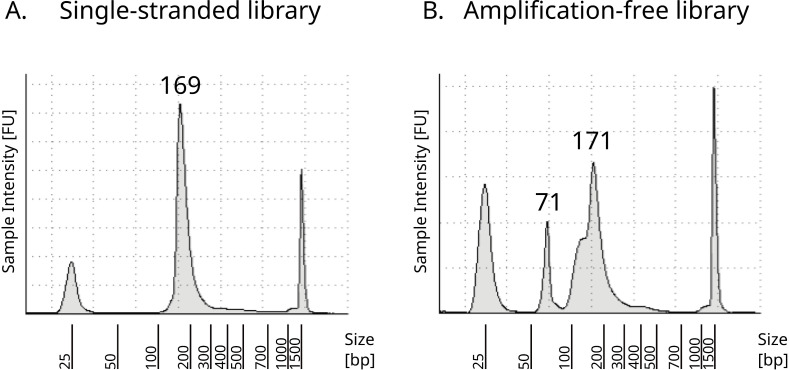
TapeStation results from the libraries for the standard single-stranded protocols **(A)** (Standard D1000 tape) and amplification-free **(B)** (High Sensitivity D1000 Tape). Peaks at 25 bp (basepair) and 1500 bp are standard Tapestation markers. For the amplification-free libraries, adapter dimers have been detected in addition to the expected library peak: an extra peak can be seen around ~ 71 bp, and a disruption of the distribution of DNA fragment lengths around 130 bp, suggesting the presence of multiple adapter dimers.

During preparation of amplification-free libraries, we observed an increased presence of adapter dimers in the purified product compared to the standard single-stranded protocol (Fig 6), likely due to the increased length of the oligonucleotides used (Fig 1). At time of this work, a combination of Tapestation and Qubit quantification of libraries was performed prior to sequencing on the in-house sequencer (NextSeq 500), which has been shown to be highly successful for achieving reliable cluster densities for standard single-stranded libraries [25]. We found that the cluster density achieved for the amplification-free library was substantially lower than the standard cluster densities (84 k/mm^2^, compared to optimal cluster densities of 180-220 k/mm^2^ following Illumina recommendations). The method of pre-sequencing library quantification we used does not distinguish between sequenceable (i.e. with the correct adapter on either end of the molecule) and non-sequenceable dimers or artefacts. The majority of adapter dimers observed in the amplification-free libraries are highly likely to be of non-sequenceable type, as the amount of adapter dimers in the sequencing data (defined here as reads reduced to 0 bp after adapter trimming) is low (S2 Table in S1 File). Therefore, the issue of low cluster density can likely be mitigated by using qPCR for quantification of the library prior to sequencing, as described in the original single-stranded protocol [3]. Alternatively, adjusting the adapter amounts or an approach as followed in Karlsson et al. [15] with a random overhang added to oligo CL9_Phos_index (Fig 1) could potentially reduce the formation of adapter dimers.

## Conclusions

The amplification-free adaptation of the standard single-stranded library preparation protocol we present here successfully generated sequence data from Pleistocene, Holocene and historical samples. We find that the data is highly consistent between the amplification-free and single-stranded protocols, suggesting that PCR does not introduce major biases in the standard single-stranded library construction. Our amplification-free adaptation of the single-stranded library preparation method represents a method that, through the removal of the indexing PCR, excludes a step that is often associated with the introduction of biases. Thus, the amplification-free approach provides a more accurate representation of the original template molecules in an ancient DNA extract. We use our amplification-free protocol to generate a nuclear genome of a Pleistocene cave bear, and perform basic population clustering methods to show that this method is not expected to cause any biases in downstream analysis compared to the standard single-stranded protocol.

Our study represents a proof-of-concept study, showcasing a method for sequencing ancient and other degraded DNA without any amplification prior to sequencing. Amplification-free library preparation from degraded samples could find a range of applications both within and outside of palaeogenomics. The ability to more directly characterise DNA content of such samples has the potential to provide new insights into the properties of damaged DNA and its degradation processes. Furthermore, amplification-free library preparation can be of interest for e.g. (ancient) poolseq and metagenomics, where PCR could introduce a bias in the relative presences of alleles, individuals or species present in the input material [41]. Similarly, the study of individual genome copy number variation and structural variants could benefit from the amplification-free method.

One further exciting future research avenue would be to implement the protocol presented here for the recovery and sequencing of ancient RNA. The field of ancient RNA is still emerging, and our protocol could allow for a direct characterisation of ancient RNA molecules and RNA specific damage occurring over time. As amplification could be problematic for comparing expression levels across different genes, the use of an amplification-free protocol specifically designed for ancient samples could mitigate some of these issues.

## Supporting information

S1 File S1 Fig. DNA yield (total and endogenous fraction in grey and black, respectively) from 14 cave bear petrous bones, showcasing the potential genome recovery from Pleistocene samples. For each sample, 50 mg bone powder was sampled from the otic capsule of the petrous bone as described in Alberti et al. [7]. DNA was extracted using the methods of Dabney et al. [2] and quantified using a Qubit fluorometer high sensitivity assay of 1µl of the DNA extracted. The estimated concentration was multiplied by the 25 μl elution volume to obtain an estimate of the total DNA yield. Single stranded libraries [3] were prepared from the extracts and low level sequencing carried out on an Illumina NextSeq platform [25]. The resulting data were mapped to the reference genome of the polar bear using the procedure described in Barlow et al. [32,33] to provide an estimate of endogenous content. S2 Fig. Nucleotide misincorporation and read length plots for deeper sequencing of sample O34-16, for the standard single-stranded library protocol (SS; red) and the amplification-free protocol (AF; grey). Plots were generated using AMBER [29] with default parameters. (A) mismatch frequency and (B) nucleotide misincorporation at each position in the read, (C) read length and (D) depth in 1kb windows. Note that the amplification-free library was sequenced using a 75bp single-end sequencing method, and the single-stranded library using 75bp paired-end method. This leads to an accumulation of all fragments that were > 75bp at a read length of 74-75bp for the amplification-free library which can be seen in panel C (AF; grey). S3 Fig. Nucleotide misincorporation plots for all samples, for the standard single-stranded library protocol (SS) and the amplification-free protocol (AF). Plots were generated using AMBER [29] with default parameters. Panels for each figure are the same as defined in S2 Fig legend. S1 Table. Wetlab information. S2 Table. Sequence data statistics. S3 Table. Subsampled sequence data statistics. S4 Table. Published cave bear datasets included in population clustering analysis. S5 Table. Oligonucleotides used in this study. Lowercase bases indicate index sequence. For more information, please refer to the single-stranded protocol (Gansauge & Meyer 2013). S1 Protocol. S1 Data. Raw qPCR results (csv file).
(ZIP)

## References

[1] Shapiro B, Hofreiter M. A paleogenomic perspective on evolution and gene function: new insights from ancient DNA. Science. 2014;343(6169):1236573. doi: 10.1126/science.1236573 24458647

[2] Dabney J, Knapp M, Glocke I, Gansauge M-T, Weihmann A, Nickel B, et al. Complete mitochondrial genome sequence of a Middle Pleistocene cave bear reconstructed from ultrashort DNA fragments. Proc Natl Acad Sci U S A. 2013;110(39):15758–63. doi: 10.1073/pnas.1314445110 24019490 PMC3785785

[3] Gansauge M-T, Meyer M. Single-stranded DNA library preparation for the sequencing of ancient or damaged DNA. Nat Protoc. 2013;8(4):737–48. doi: 10.1038/nprot.2013.038 23493070

[4] Meyer M, Kircher M, Gansauge M-T, Li H, Racimo F, Mallick S, et al. A high-coverage genome sequence from an archaic Denisovan individual. Science. 2012;338(6104):222–6. doi: 10.1126/science.1224344 22936568 PMC3617501

[5] Kapp JD, Green RE, Shapiro B. A fast and efficient single-stranded genomic library preparation method optimized for ancient DNA. J Hered. 2021;112(3):241–9. doi: 10.1093/jhered/esab012 33768239 PMC8141684

[6] Pinhasi R, Fernandes D, Sirak K, Novak M, Connell S, Alpaslan-Roodenberg S, et al. Optimal ancient DNA yields from the inner ear part of the human petrous bone. PLoS One. 2015;10(6):e0129102. doi: 10.1371/journal.pone.0129102 26086078 PMC4472748

[7] Alberti F, Gonzalez J, Paijmans JLA, Basler N, Preick M, Henneberger K, et al. Optimized DNA sampling of ancient bones using computed tomography scans. Mol Ecol Resour. 2018;18(6):1196–208. doi: 10.1111/1755-0998.12911 29877032

[8] Damgaard PB, Margaryan A, Schroeder H, Orlando L, Willerslev E, Allentoft ME. Improving access to endogenous DNA in ancient bones and teeth. Sci Rep. 2015;5:11184. doi: 10.1038/srep11184 26081994 PMC4472031

[9] Aird D, Ross MG, Chen W-S, Danielsson M, Fennell T, Russ C, et al. Analyzing and minimizing PCR amplification bias in Illumina sequencing libraries. Genome Biol. 2011;12(2):R18. doi: 10.1186/gb-2011-12-2-r18 21338519 PMC3188800

[10] Dabney J, Meyer M. Length and GC-biases during sequencing library amplification: a comparison of various polymerase-buffer systems with ancient and modern DNA sequencing libraries. Biotechniques. 2012;52(2):87–94. doi: 10.2144/000113809 22313406

[11] Star B, Hansen MH, Skage M, Bradbury IR, Godiksen JA, Kjesbu OS, et al. Preferential amplification of repetitive DNA during whole genome sequencing library creation from historic samples. STAR: Sci Technol Archaeol Res. 2016;2(1):36–45. doi: 10.1080/20548923.2016.1160594

[12] Kozarewa I, Ning Z, Quail MA, Sanders MJ, Berriman M, Turner DJ. Amplification-free Illumina sequencing-library preparation facilitates improved mapping and assembly of (G+C)-biased genomes. Nat Methods. 2009;6(4):291–5. doi: 10.1038/nmeth.1311 19287394 PMC2664327

[13] Quail MA, Kozarewa I, Smith F, Scally A, Stephens PJ, Durbin R, et al. A large genome center’s improvements to the Illumina sequencing system. Nat Methods. 2008;5(12):1005–10. doi: 10.1038/nmeth.1270 19034268 PMC2610436

[14] Kozarewa I, Turner DJ. Amplification-free library preparation for paired-end Illumina sequencing. Methods Mol Biol. 2011;733:257–66. doi: 10.1007/978-1-61779-089-8_18 21431776

[15] Karlsson K, Sahlin E, Iwarsson E, Westgren M, Nordenskjöld M, Linnarsson S. Amplification-free sequencing of cell-free DNA for prenatal non-invasive diagnosis of chromosomal aberrations. Genomics. 2015;105(3):150–8. doi: 10.1016/j.ygeno.2014.12.005 25543032

[16] Bennett EA, Massilani D, Lizzo G, Daligault J, Geigl E-M, Grange T. Library construction for ancient genomics: single strand or double strand? Biotechniques. 2014;56(6):289–90, 292–6, 298, passim. doi: 10.2144/000114176 24924389

[17] Barlow A, Fortes GG, Dalén L, Pinhasi R, Gasparyan B, Rabeder G, et al. Massive influence of DNA isolation and library preparation approaches on palaeogenomic sequencing data. 2016. doi: 10.1101/075911

[18] Barlow A, Hartmann S, Gonzalez J, Hofreiter M, Paijmans JLA. Consensify: a method for generating pseudohaploid genome sequences from palaeogenomic datasets with reduced error rates. 2018. doi: 10.1101/498915 PMC7017230 31906474

[19] Gansauge M-T, Gerber T, Glocke I, Korlevic P, Lippik L, Nagel S, et al. Single-stranded DNA library preparation from highly degraded DNA using T4 DNA ligase. Nucleic Acids Res. 2017;45(10):e79. doi: 10.1093/nar/gkx033 28119419 PMC5449542

[20] R Core Team. R: A language and environment for statistical computing. 2013. Available from: http://www.R-project.org/

[21] Ritz C, Spiess A-N. qpcR: an R package for sigmoidal model selection in quantitative real-time polymerase chain reaction analysis. Bioinformatics. 2008;24(13):1549–51. doi: 10.1093/bioinformatics/btn227 18482995

[22] Rohland N, Siedel H, Hofreiter M. Nondestructive DNA extraction method for mitochondrial DNA analyses of museum specimens. Biotechniques. 2004;36(5):814–6, 818–21. doi: 10.2144/04365ST05 15152601

[23] Paijmans JLA, Barlow A, Henneberger K, Fickel J, Hofreiter M, Foerster DWG. Ancestral mitogenome capture of the Southeast Asian banded linsang. PLoS One. 2020;15(6):e0234385. doi: 10.1371/journal.pone.0234385 32603327 PMC7326216

[24] Straube N, Lyra ML, Paijmans JLA, Preick M, Basler N, Penner J, et al. Successful application of ancient DNA extraction and library construction protocols to museum wet collection specimens. Mol Ecol Resour. 2021;21(7):2299–315. doi: 10.1111/1755-0998.13433 34036732

[25] Paijmans J, Baleka S, Henneberger K, Taron U, Trinks A, Westbury M. Sequencing single-stranded libraries on the Illumina NextSeq 500 platform. ArXiv. 2017;1711.11004.

[26] Martin M. Cutadapt removes adapter sequences from high-throughput sequencing reads. EMBnet.journal. 2011;17:10–2.

[27] Li H, Durbin R. Fast and accurate short read alignment with Burrows-Wheeler transform. Bioinformatics. 2009;25(14):1754–60. doi: 10.1093/bioinformatics/btp324 19451168 PMC2705234

[28] Li H, Handsaker B, Wysoker A, Fennell T, Ruan J, Homer N, et al. The sequence alignment/Map format and SAMtools. Bioinformatics. 2009;25(16):2078–9. doi: 10.1093/bioinformatics/btp352 19505943 PMC2723002

[29] Dolenz S, van der Valk T, Jin C, Oppenheimer J, Sharif MB, Orlando L, et al. Unravelling reference bias in ancient DNA datasets. Bioinformatics. 2024;40(7):btae436. doi: 10.1093/bioinformatics/btae436 38960861 PMC11254355

[30] Wickham H, Averick M, Bryan J, Chang W, McGowan L, François R, et al. Welcome to the Tidyverse. JOSS. 2019;4(43):1686. doi: 10.21105/joss.01686

[31] Magoč T, Salzberg SL. FLASH: fast length adjustment of short reads to improve genome assemblies. Bioinformatics. 2011;27(21):2957–63. doi: 10.1093/bioinformatics/btr507 21903629 PMC3198573

[32] Barlow A, Cahill JA, Hartmann S, Theunert C, Xenikoudakis G, Fortes GG, et al. Partial genomic survival of cave bears in living brown bears. Nat Ecol Evol. 2018;2(10):1563–70. doi: 10.1038/s41559-018-0654-8 30150744 PMC6590514

[33] Barlow A, Paijmans JLA, Alberti F, Gasparyan B, Bar-Oz G, Pinhasi R, et al. Middle Pleistocene genome calibrates a revised evolutionary history of extinct cave bears. Curr Biol. 2021;31(8):1771-1779.e7. doi: 10.1016/j.cub.2021.01.073 33592193

[34] Korneliussen TS, Albrechtsen A, Nielsen R. ANGSD: analysis of next generation sequencing data. BMC Bioinformatics. 2014;15(1):356. doi: 10.1186/s12859-014-0356-4 25420514 PMC4248462

[35] Paradis E, Claude J, Strimmer K. APE: analyses of phylogenetics and evolution in R language. Bioinformatics. 2004;20(2):289–90. doi: 10.1093/bioinformatics/btg412 14734327

[36] Revell LJ. phytools: an R package for phylogenetic comparative biology (and other things). Methods Ecol Evol. 2011;3(2):217–23. doi: 10.1111/j.2041-210x.2011.00169.x

[37] Sheng G-L, Basler N, Ji X-P, Paijmans JLA, Alberti F, Preick M, et al. Paleogenome reveals genetic contribution of extinct giant panda to extant populations. Curr Biol. 2019;29(10):1695-1700.e6. doi: 10.1016/j.cub.2019.04.021 31080081

[38] Jónsson H, Ginolhac A, Schubert M, Johnson PLF, Orlando L. mapDamage2.0: fast approximate Bayesian estimates of ancient DNA damage parameters. Bioinformatics. 2013;29(13):1682–4. doi: 10.1093/bioinformatics/btt193 23613487 PMC3694634

[39] Baryshnikov GF, Puzachenko AYu. Craniometrical variability in the cave bears (Carnivora, Ursidae): Multivariate comparative analysis. Quaternary International. 2011;245(2):350–68. doi: 10.1016/j.quaint.2011.02.035

[40] Stiller M, Molak M, Prost S, Rabeder G, Baryshnikov G, Rosendahl W, et al. Mitochondrial DNA diversity and evolution of the Pleistocene cave bear complex. Quaternary International. 2014;339–340:224–31. doi: 10.1016/j.quaint.2013.09.023

[41] Korvigo I, Igolkina AA, Kichko AA, Aksenova T, Andronov EE. Be aware of the allele-specific bias and compositional effects in multi-template PCR. PeerJ. 2022;10:e13888. doi: 10.7717/peerj.13888 36061756 PMC9438772

