# Supplementary material for: A method for PCR-free library preparation for sequencing palaeogenomes
Source: PLoS One. 2025 Mar 19;20(3):e0319573. doi: 10.1371/journal.pone.0319573 (PMC11922524; doi:10.1371/journal.pone.0319573)
Supplement: S1 File — For each sample, 50 mg bone powder was sampled from the otic capsule of the petrous bone as described in Alberti et al. [7]. DNA was extracted using the methods of Dabney et al. [2] and quantified using a Qubit fluorometer high sensitivity assay of 1µl of the DNA extracted. The estimated concentration was multiplied by the 25 μl elution volume to obtain an estimate of the total DNA yield. Single stranded libraries [3] were prepared from the extracts and low level sequencing carried out on an Illumina NextSeq platform [25]. The resulting data were mapped to the reference genome of the polar bear using the procedure described in Barlow et al. [32,33] to provide an estimate of endogenous content. S2 Fig. Nucleotide misincorporation and read length plots for deeper sequencing of sample O34-16, for the standard single-stranded library protocol (SS; red) and the amplification-free protocol (AF; grey). Plots were generated using AMBER [29] with default parameters. (A) mismatch frequency and (B) nucleotide misincorporation at each position in the read, (C) read length and (D) depth in 1kb windows. Note that the amplification-free library was sequenced using a 75bp single-end sequencing method, and the single-stranded library using 75bp paired-end method. This leads to an accumulation of all fragments that were > 75bp at a read length of 74-75bp for the amplification-free library which can be seen in panel C (AF; grey). S3 Fig. Nucleotide misincorporation plots for all samples, for the standard single-stranded library protocol (SS) and the amplification-free protocol (AF). Plots were generated using AMBER [29] with default parameters. Panels for each figure are the same as defined in S2 Fig legend. S1 Table. Wetlab information. S2 Table. Sequence data statistics. S3 Table. Subsampled sequence data statistics. S4 Table. Published cave bear datasets included in population clustering analysis. S5 Table. Oligonucleotides used in this study. Lowercase bases indicate index sequenc [file pone.0319573.s001.zip › S1_protocol.pdf]

Protocol for:

A method for PCR-free library preparation for sequencing palaeogenomes

Kirstin Henneberger, Axel Barlow, Federica Alberti, Michaela Preick, Silviu Constantin, Doris Döppes, Wilfried Rosendahl, Michael Hofreiter, Johanna L.A. Paijmans

REAGENTS AND MATERIALS

Chemicals:

Nuclease free water

HPLC-grade water

CircLigase II ssDNA Ligase (Epicentre), including 10X Reaction Buffer and 50 mM MnCl2

Endonuclease VIII

Afu UDG

FastAP

PEG-4000 (50 %)

5 M NaCl

0.5 M EDTA (pH 8.0)

1 M Tris-HCl (pH 8.0)

20X SSC buffer

Adapter oligo CL78 (100 µM)

Dynabeads MyOne Streptavidin C1

SDS solution 20% (wt/col)

Klenow Fragment (Thermo Scientific), including 10X Reaction Buffer

dNTP mix

pUC19 vector

Extension primer CL9_Phos_index

T4 DNA Ligase Buffer (10x)

T4 DNA Ligase

Tween 20

AccuPrime Pfx Reaction Mix (10x)

AccuPrime Pfx Polymerase

CL72 (100 µM)

Gesaffelstein (100 µM)

CL104 (100 µM)

IS5 (100 µM)

CL9_Phos_index (100 µM)

Double-stranded adapter long, strand 1 = CL53 (500 µM)

Double-stranded adapter long, strand 2 incl. index = CL73_index (500 µM)

MinElute PCR Purification Kit

Buffer setup:

Tween 20 (1 % vol/vol): diluted in Nuclease free water

TE buffer: 49.4 ml of HPLC-grade water, 500 µl of 1 M Tris-HCl (pH 8.0) and 100 µl of 0.5 M EDTA (pH 8.0)

TET buffer: 49.375 ml of HPLC-grade water, 500 µl of 1 M Tris-HCl, 100 µl of 0.5 M EDTA and 25 µl of Tween 20

Bead-binding buffer: 7.63 ml of HPLC-grade water, 2 ml of 5 M NaCl, 100 µl of 1 M Tris-HCl (pH 8.0), 20 µl of 0.5 M EDTA (pH 8.0), 5 µl of Tween 20 and 250 µl of 20% (wt/vol) SDS. Attention: Prepare this buffer by combining all reagents except for SDS. Add SDS just before using this buffer. Shelf life of this buffer after SDS addition is very short. Make sure that the stock SDS has not precipitated, otherwise heat it gently up to dissolve it.

Wash buffer A: 47.125 ml of water, 1 ml of 5 M NaCl, 500 µl of 1 M Tris-HCl (pH 8.0), 100 µl of 0.5 M EDTA (pH 8.0), 25 µl of Tween 20 and 1.25 ml of 20% (wt/vol) SDS

Wash buffer B: 48.375 ml of water, 1 ml of 5 M NaCl, 500 µl of 1 M Tris-HCl (pH 8.0), 100 µl of 0.5 M EDTA (pH 8.0) and 25 µl of Tween 20

Stringency wash buffer: 49.5 ml of water, 250 µl of 20% (wt/vol) SDS and 250 µl of 20X SSC buffer

Stop solution: 98 µl of 0.5 M EDTA (pH 8.0) and 2 µl of Tween 20

Consumables:

LoBind tubes, 1.5 ml

LoBind tubes, 0.5 ml

PCR strip tubes

PCR plate and PCR plate foil

Thermal cyclers with lid heating; 0.2-ml PCR tubes and 0.5-ml tubes Microcentrifuges

Plate centrifuge for 96-well PCR plates

PCR-Cooler 0.2 ml and 2.0 ml

Magnetic rack for 1.5-ml tubes

Rotator for 1.5-ml tubes

Vortex mixer

Cooling ThermoMixer

qPCR machine

Tapestation instrument, Tapestation tapes and reagents (buffer, ladder)

Illumina sequencing instrument (e.g. NextSeq)

MinElute

Oligos:

For full list of oligo sequences, see Supporting Table S5.

Adapter oligo CL78 (10 µM), diluted in TE buffer

IS5 (10 µM), diluted in TE buffer

CL104 (0.1 µM), diluted in TET

Double-stranded adapter long mix_index (100 µM): Set up the following hybridization reaction mix in a PCR tube, using a unique combination of indexing primers for each sample. The preparation of a master mix without the double-stranded adapter long, strand 2 including the index (= CL73_index) is recommended to avoid pipetting too small amounts of some reagents. For one hybridization reaction combine 1.9 µl of TE buffer, 0.1 µl of 5M NaCl, 4 µl of 500 µM oligonucleotide CL53 and 4 µl of 500 µM oligonucleotide CL73_index. Incubate the reaction mixture in a thermal cycler for 10 s at 95 °C and slowly decrease the temperature at the rate of 0.1 °C per second until reaching 14 °C. Add 10 µl TE buffer to each reaction to obtain a concentration of 100 µM of double-stranded adapter long mix_index in a total volume of 20 µl. The resulting solution can be stored at − 20 °C for at least 1 year.

PROCEDURE

STAGE 1: Uracil excision and DNA cleavage at abasic sites

1. For each sample, prepare the following reaction mixture with a total volume of 44 µl in 0.2-ml PCR tubes. Mix by flicking the tubes with a finger and spin the tubes briefly in a microcentrifuge.

Critical Step: Always include a blank control using water instead of DNA extract. UDG treatment is optional, but endonuclease VIII should be included because it cleaves DNA at abasic sites if present.

Reagent	Final concentration in reaction in this step (in step 5)	Volume (µl) per sample
Nuclease free water	-	11
CircLigase Buffer II (10x)	1.8x (1x in stage 3)	8
MnCl2 (50 mM)	4.5 mM (2.5 mM in stage 3)	4
Endonuclease VIII (10 U/µl)	0.11 U/µl	0.5
Afu UDG (2 U/µl)	0.02 U/µl	0.5

2. Incubate the reactions in a thermal cycler with a heated lid for 1 h at 37 °C.

STAGE 2: Dephosphorylation and heat denaturation

3. Add 1 µl of FastAP (1 U) to each reaction mixture prepared in Step 1 and mix by flicking the tubes with your finger. Spin the tubes briefly in a microcentrifuge. The total reaction volume is now 45 µl.
4. Incubate the reactions in a thermal cycler with a heated lid for 10 min at 37 °C, and then at 95 °C for 2 min. While the thermal cycler is still at 95 °C, quickly transfer the tubes into a freezer cooling block. Let the reaction mix cool down for at least 1 min. Spin the tubes briefly in a microcentrifuge and place them in a tube rack at room temperature.

STAGE 3: Ligation of the first adapter

5. Add the following components to the reaction mixtures to obtain a final reaction volume of 80 µl. Mix the contents of the tubes by vortexing before adding CircLigase II; mix vigorously by flicking the tube with a finger thereafter. Spin the tubes briefly in a microcentrifuge.

Reagent	Final concentration in 80 µl in step 3.5	Volume (µl) per sample
PEG-4000 (50 %)	20 %	32
Adapter oligo CL78 (10 µM)	0.125 µM	1
CircLigase II (100 U/µl)	2.5 U/µl	2

Critical Step: PEG solution is highly viscous. Pipette it slowly and ensure that all components are properly mixed.

6. Incubate the reaction mixtures in a thermal cycler with a heated lid for 1 h at 60 °C.
7. Add 2 µl of stop solution to each reaction mixture. Mix the contents by vortexing and spin the tubes in a microcentrifuge.

PAUSE POINT Ligation products can be stored safely at − 20 °C for several days.

STAGE 4: Immobilization of ligation products on beads

Critical Step: It is important that the beads do not sediment at the bottom of the tubes. You should vortex them every couple of minutes, or mix in the thermomixer (100% short mix function).

8. Resuspend the stock of MyOne C1 beads by vortexing. For each sample, transfer 20 µl of the bead suspension into a 1.5 ml tube (e.g., 80 µl for four reactions). Pellet the beads using a magnetic rack, discard the supernatant and wash the beads twice with 500 µl of bead-binding buffer. Resuspend the beads in a volume of bead-binding buffer corresponding to the number of samples times 250 µl (e.g., 1 ml for four samples). Per sample, transfer an aliquot of 250 µl of bead suspension to a 1.5 ml tube.
9. Incubate the ligation reactions from Step 7 for 1 min at 95 °C in a thermal cycler with a heated lid. While the thermal cycler is still at 95 °C, quickly transfer the tubes into a freezer cooling block. Let the reaction mixture cool down for at least 1 min. Spin the tubes briefly in a microcentrifuge and add the ligation reactions to the bead suspensions prepared in Step 8.
10. Rotate the tubes for 20 min at room temperature.
11. Spin the tubes briefly in a microcentrifuge. Pellet the beads using a magnetic rack and discard the supernatant. Wash the beads once with 200 µl of wash buffer A and once with 200 µl of wash buffer B (see Equipment Setup for instructions on how to perform bead washes).

STAGE 5: Primer annealing and extension

Critical Step: Make sure that the thermomixer and thermal cycler are already preheated to the desired temperatures in the corresponding steps.

12. Prepare a master mix for the required number of reactions (47 µl per reaction).

Reagent	Final concentration in reaction	Volume (µl) per sample
Nuclease free water	-	41.6
Klenow Buffer (10x)	1x	5

dNTP mix (25 mM each)	200 µM each	0.4

13. Pellet the beads using a magnetic rack and discard the wash buffer. Add the 47 µl reaction mixture to the pelleted beads, add 1 µl CL9_Phos_index (100 µM) (with a final concentration of 2 µM in the reaction) and resuspend the beads by vortexing. Incubate the tubes in a thermal cycler for 2 min at 65 °C (leave the lid of the thermal cycler open). Place the tubes in a freezer cooling block for 1 min, and then immediately transfer the tubes to a thermomixer preheated to 25 °C. While the tubes are placed on the thermomixer, add 2 µl of Klenow Fragment (20 U) to each reaction mixture.
14. Incubate the reaction mixtures in the thermomixer for 5 min at 25 °C, followed by 25 min at 35 °C. Keep the beads suspended during incubation by mixing every minute.
15. Spin the tubes briefly in a microcentrifuge. Pellet the beads using a magnetic rack and discard the supernatant. Wash the beads once with 200 µl of wash buffer A. Resuspend the beads in 100 µl of stringency wash buffer and incubate the bead suspensions for 3 min at 45 °C in a thermomixer. Pellet the beads using a magnetic rack and discard the supernatant. Wash the beads once with 200 µl of wash buffer B.

STAGE 6: Ligation of the second adapter and library elution

16. Prepare a master mix for the required number of reactions (96 µl per reaction).

Reagent	Final concentration in 100 µl	Volume (µl) per sample
Nuclease free water	-	73.5
T4 DNA Ligase Buffer (10x)	1x	10
PEG-4000 (50 %)	5 %	10
Tween 20 (1 %)	0.025 %	2.5

17. Pellet the beads using a magnetic rack and discard the wash buffer. Add 96 µl of the reaction mixture from Step 16 to the pelleted beads and add 2 µl of double-stranded adapter long mix_index (100 µM) (with a final concentration of 2 µM in the reaction) and resuspend the beads by vortexing. Add 2 µl of T4 DNA Ligase (10 U). Mix the contents briefly by vortexing.
18. Incubate the reaction mixtures for 1 h at 22 °C in a thermomixer. Keep the beads suspended during incubation by mixing every minute.
19. Pellet the beads using a magnetic rack and discard the supernatant. Wash the beads with wash buffer A, stringency wash buffer (with incubation at 45 °C) and wash buffer B exactly as described in Step 15.
20. Pellet the beads using a magnetic rack and discard the supernatant. Add 25 µl of TET buffer to the pelleted beads, resuspend the beads by vortexing and transfer the bead suspension to 0.2-ml PCR strip tubes. Spin the tubes briefly in a microcentrifuge.
21. Incubate the bead suspensions for 1 min at 95 °C in a thermal cycler with a heated lid. Immediately transfer the PCR strip tubes to a 96-well magnetic rack. Transfer the supernatant, which contains the library molecules, to a fresh tube.

PAUSE POINT Ligation products can be stored safely at − 20 °C for several weeks.

STAGE 7: Preparation of double-stranded library molecules

22. Prepare a master mix for the required number of reactions (60 µl per reaction).

Reagent	Final concentration in 80 µl	Volume (µl) per sample
Nuclease free water	-	48
AccuPrime Pfx Reaction Mix (10x)	1x	8
IS5 primer (10 µM)	0.4 µM	0.8
AccuPrime Pfx Polymerase (2.5 U/µl)	0.025 U/µl	3.2
Single-stranded Library Sample	-	20

23. Incubate the reactions in a thermal cycler with the following thermal profile. Initial denaturation should be carried out at 95 °C for 2 min. Follow this by a denaturation for 15 s at 95 °C, annealing for 30 s at 60 °C and primer extension for 3 min at 68 °C.
24. Purify amplified libraries using the MinElute PCR purification kit according to the manufacturer's instructions. Elute the DNA in 10 µl of TE buffer. Repeat the elution with another 10 µl of TE buffer.
25. Determine the fragment size distributions and concentrations of the DNA libraries by running the Tapestation.

STAGE 8: Sequencing

26. For sequencing, follow the protocols and instructions for multiplex sequencing provided by Illumina. Make sure to replace the sequencing primer of the first read by the custom primer CL72 and the sequencing primer of the custom index read 2 by the custom primer Gesaffelstein. Freshly prepare a ready-to-use dilution of CL72 before sequencing by mixing 6 µl from the 100 µM stock solution with 1,994 µl of hybridization buffer (provided with the sequencing reagents).
