# Supplementary figures and images for: A method for PCR-free library preparation for sequencing palaeogenomes
Source: PLoS One. 2025 Mar 19;20(3):e0319573. doi: 10.1371/journal.pone.0319573 (PMC11922524; doi:10.1371/journal.pone.0319573)

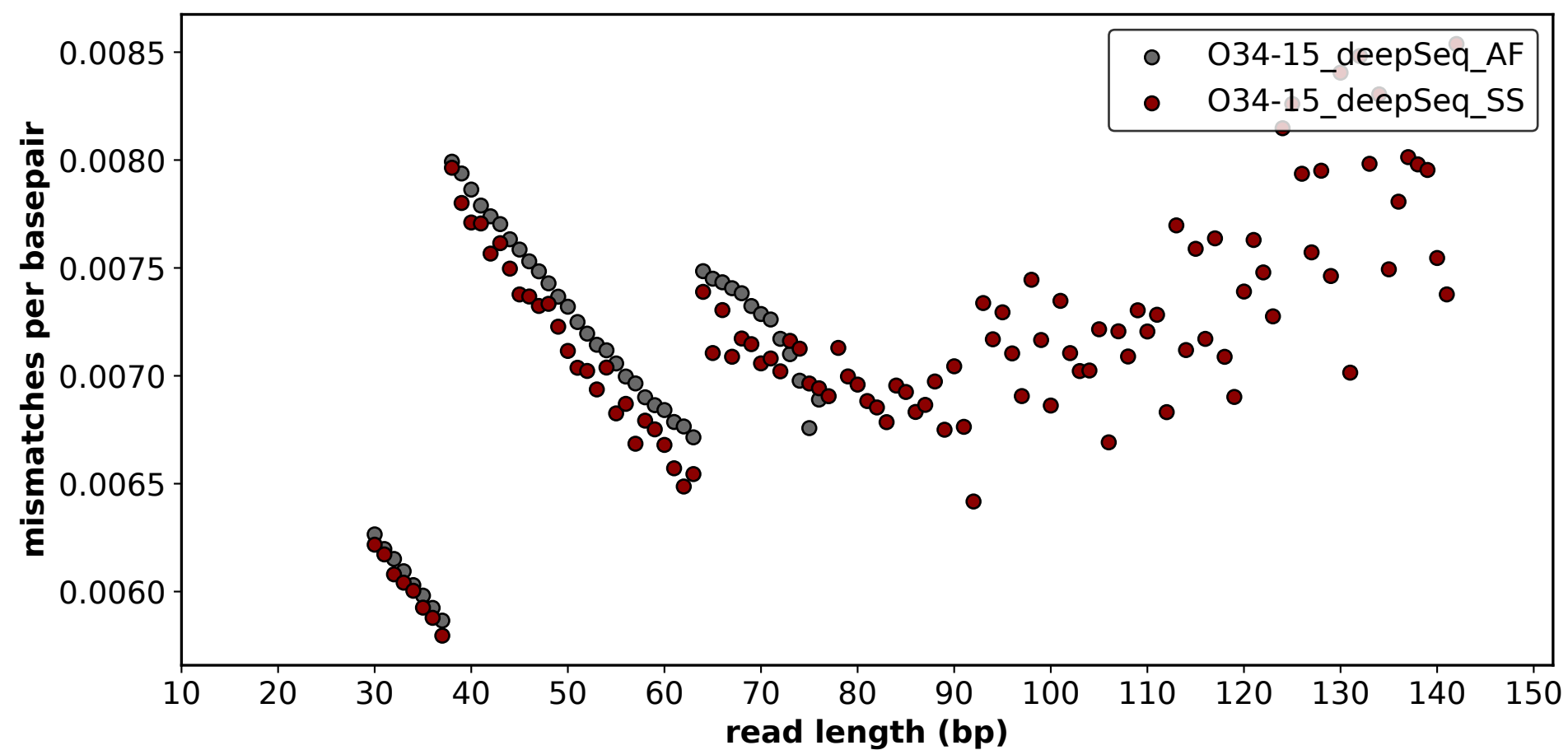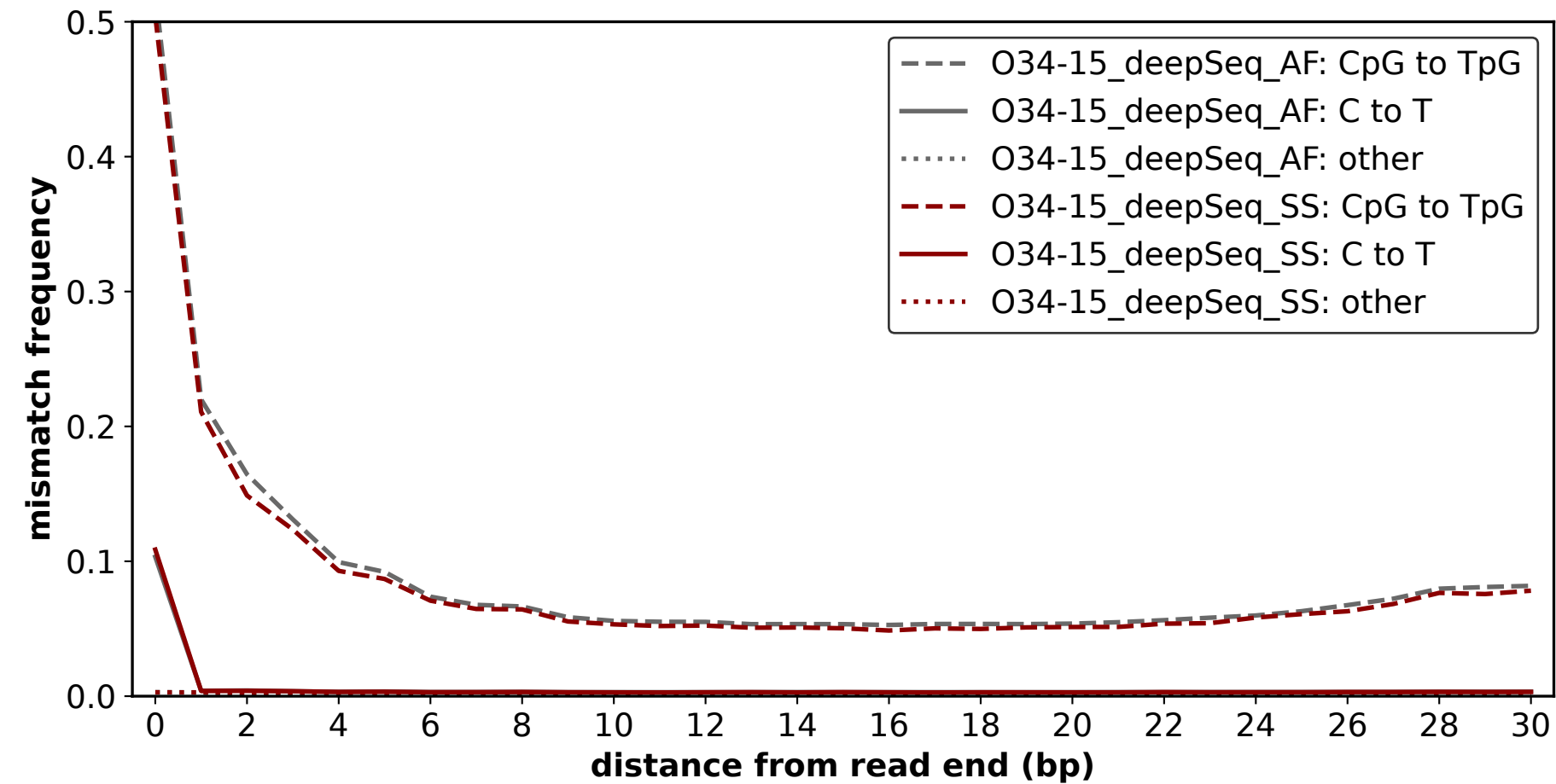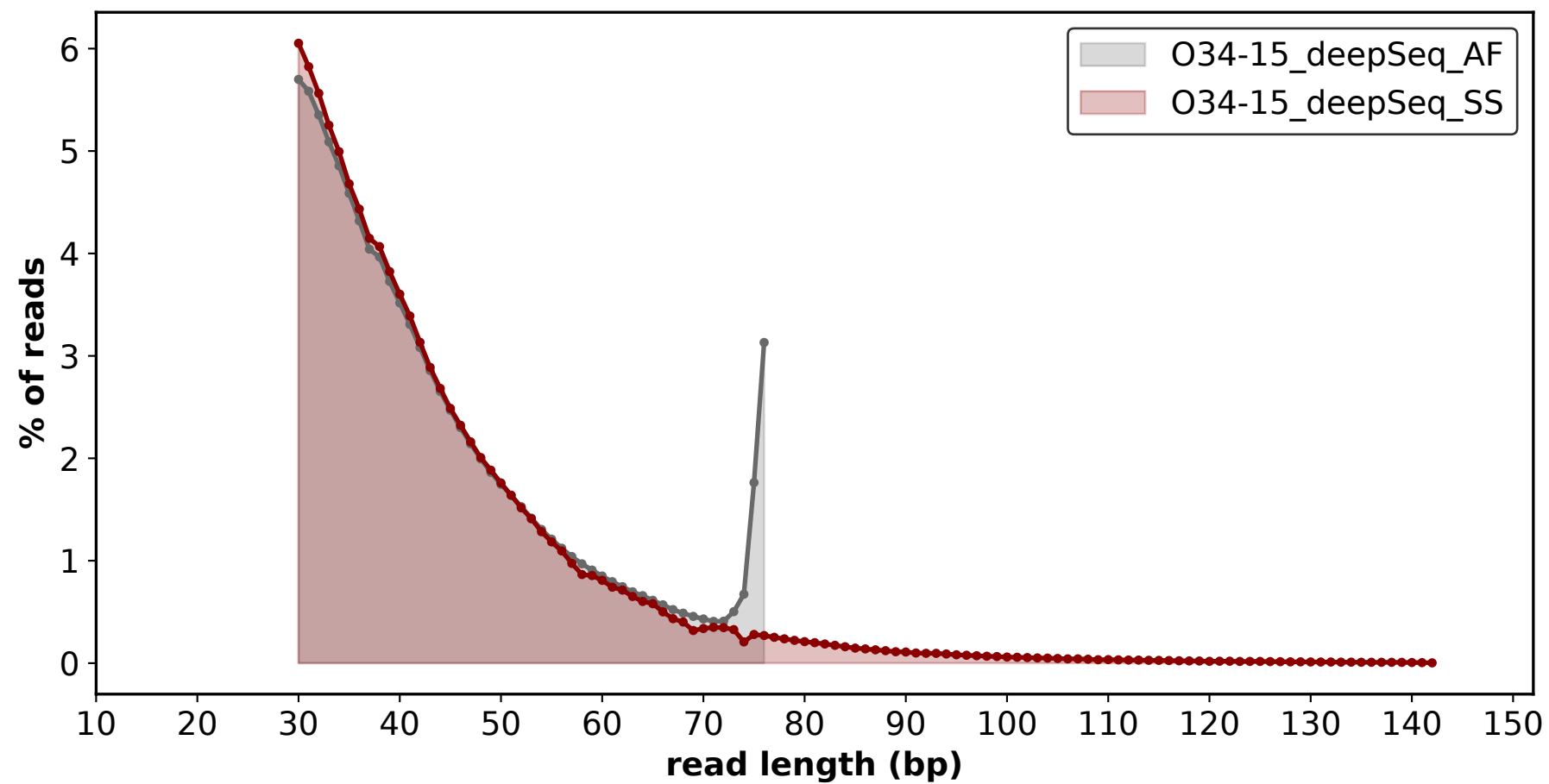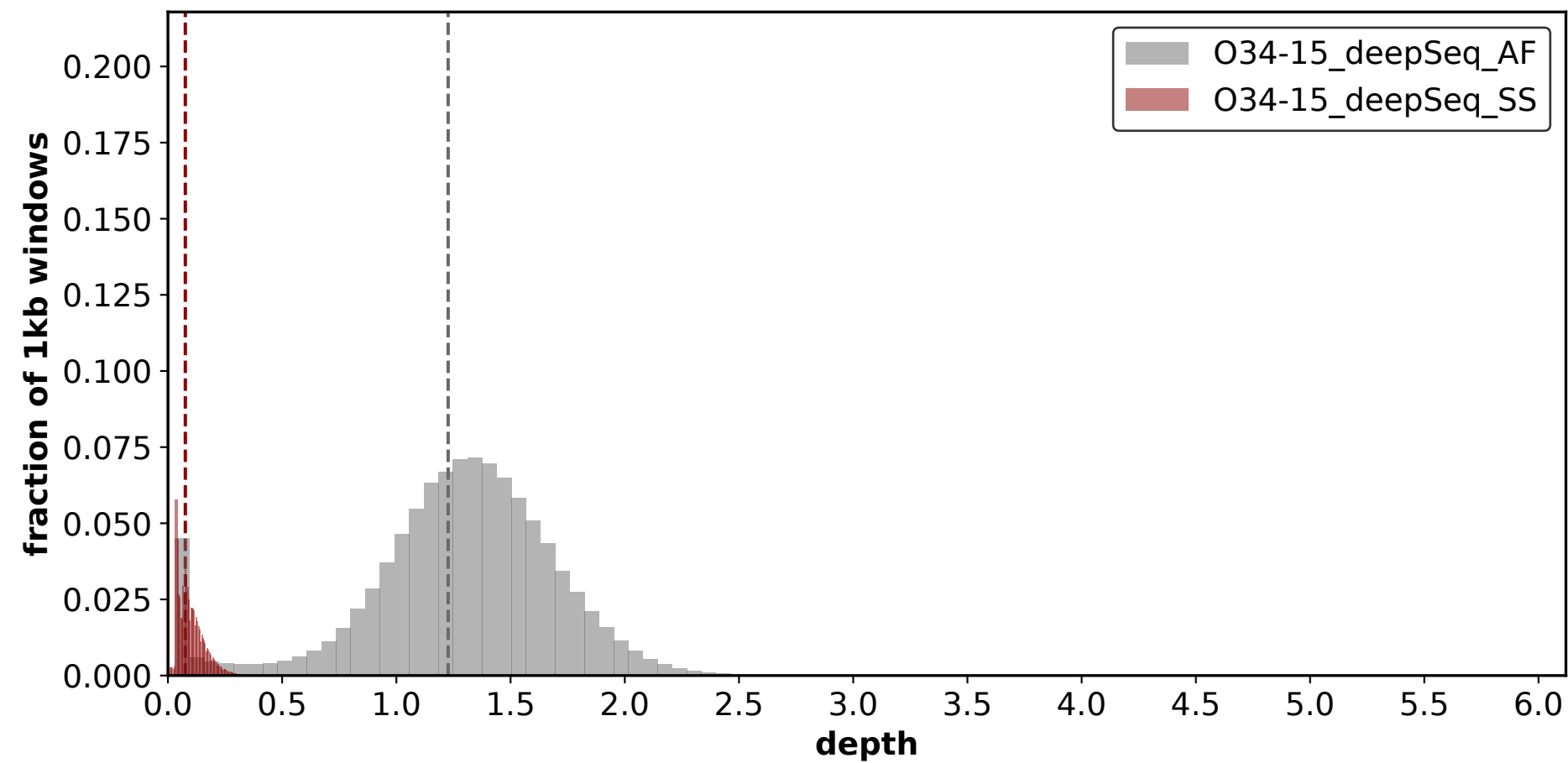

Supplement: S1 File — For each sample, 50 mg bone powder was sampled from the otic capsule of the petrous bone as described in Alberti et al. [7]. DNA was extracted using the methods of Dabney et al. [2] and quantified using a Qubit fluorometer high sensitivity assay of 1µl of the DNA extracted. The estimated concentration was multiplied by the 25 μl elution volume to obtain an estimate of the total DNA yield. Single stranded libraries [3] were prepared from the extracts and low level sequencing carried out on an Illumina NextSeq platform [25]. The resulting data were mapped to the reference genome of the polar bear using the procedure described in Barlow et al. [32,33] to provide an estimate of endogenous content. S2 Fig. Nucleotide misincorporation and read length plots for deeper sequencing of sample O34-16, for the standard single-stranded library protocol (SS; red) and the amplification-free protocol (AF; grey). Plots were generated using AMBER [29] with default parameters. (A) mismatch frequency and (B) nucleotide misincorporation at each position in the read, (C) read length and (D) depth in 1kb windows. Note that the amplification-free library was sequenced using a 75bp single-end sequencing method, and the single-stranded library using 75bp paired-end method. This leads to an accumulation of all fragments that were > 75bp at a read length of 74-75bp for the amplification-free library which can be seen in panel C (AF; grey). S3 Fig. Nucleotide misincorporation plots for all samples, for the standard single-stranded library protocol (SS) and the amplification-free protocol (AF). Plots were generated using AMBER [29] with default parameters. Panels for each figure are the same as defined in S2 Fig legend. S1 Table. Wetlab information. S2 Table. Sequence data statistics. S3 Table. Subsampled sequence data statistics. S4 Table. Published cave bear datasets included in population clustering analysis. S5 Table. Oligonucleotides used in this study. Lowercase bases indicate index sequenc [file pone.0319573.s001.zip › S2_figure_AMBERhighcov.pdf]

## A) 33961

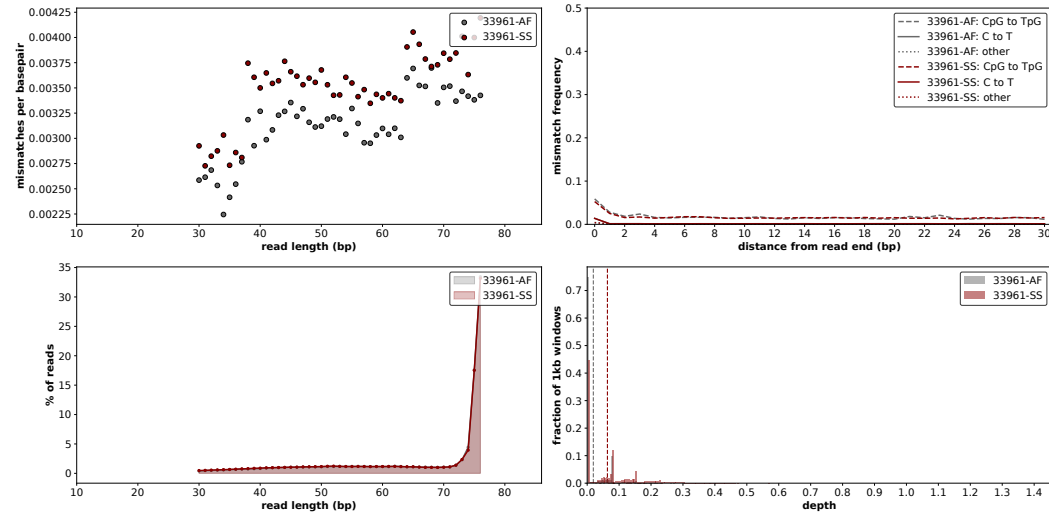

## B) 50746NB

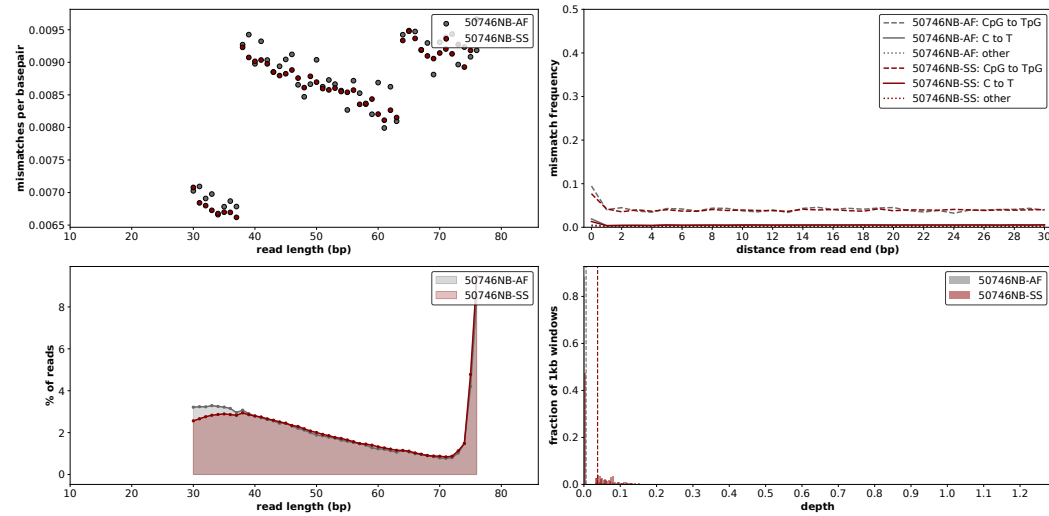

## C) 56097ST

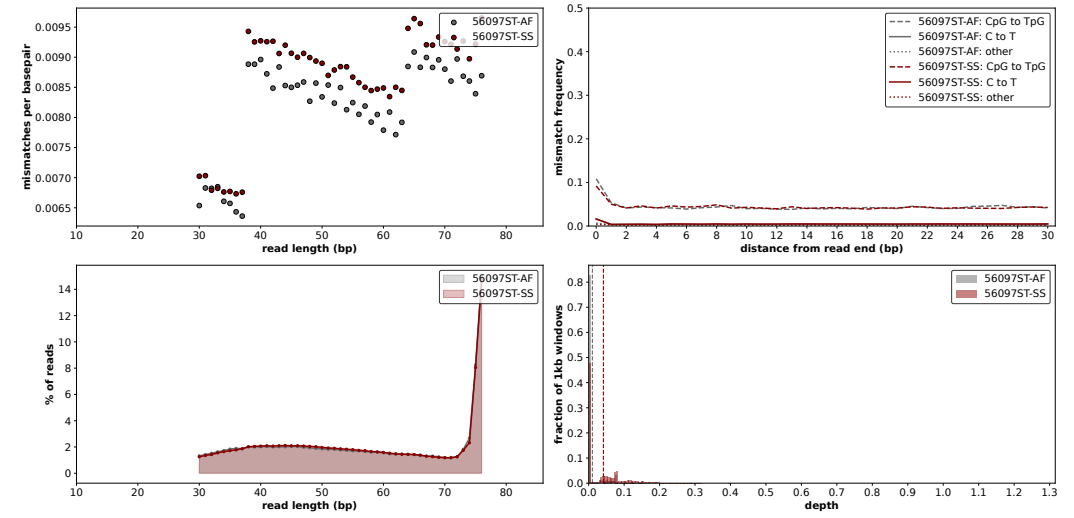

## D) 56407ST

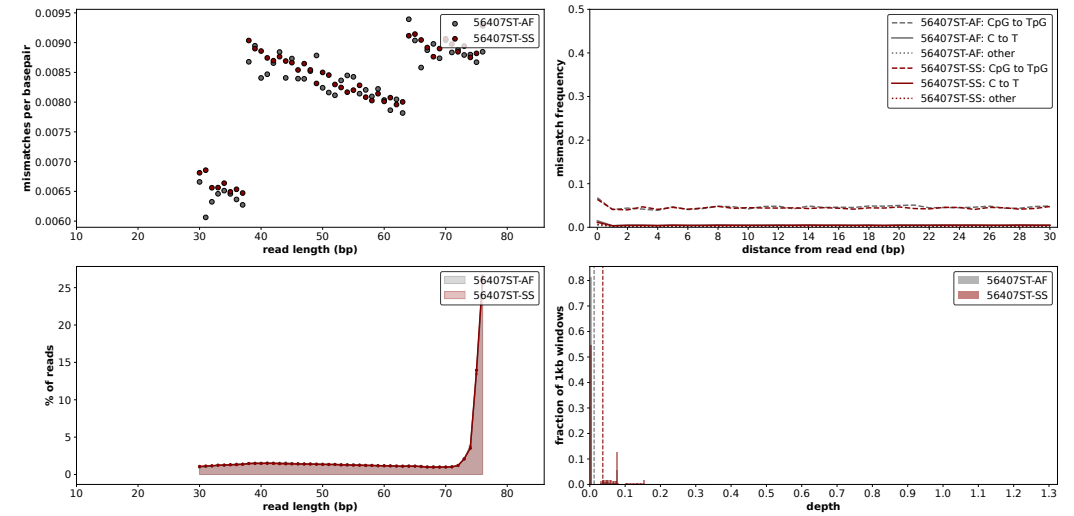

## E) 70423NB

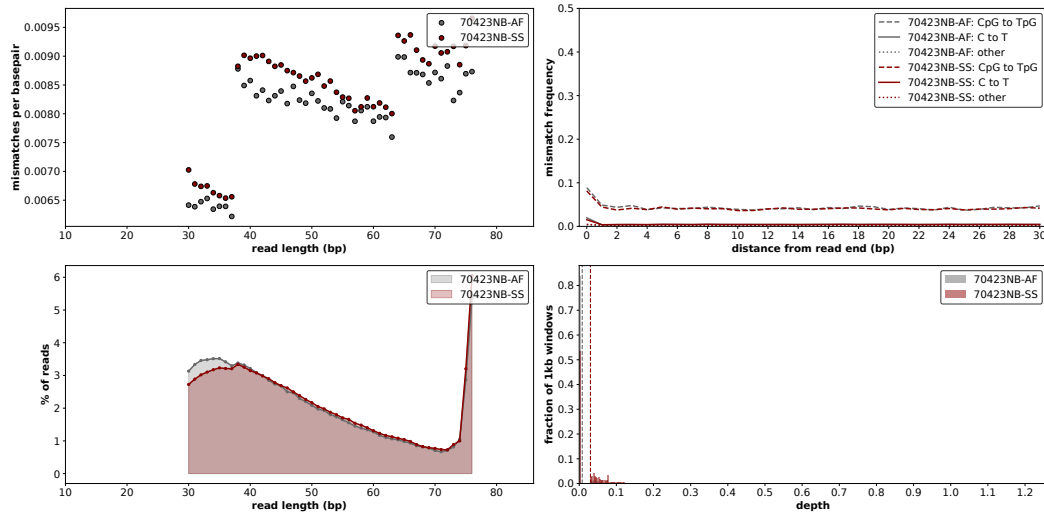

## G) PM-T6-I-II-101

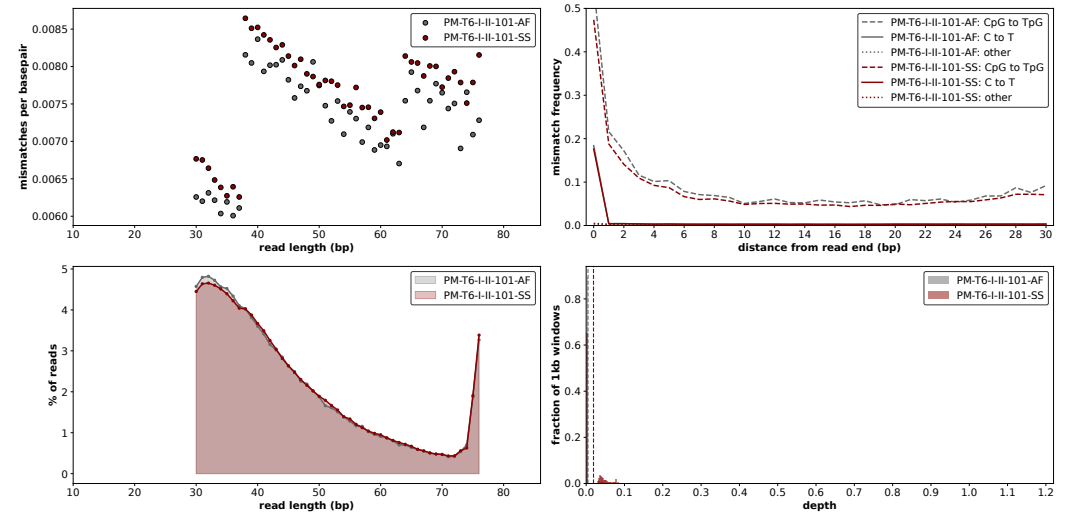

## F) PLI-19

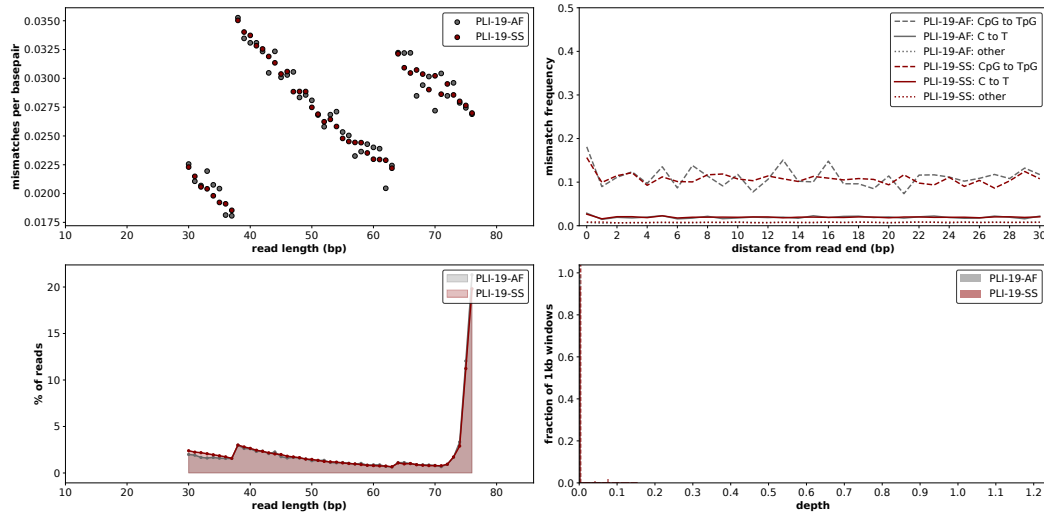

## H) PM-T6-I-II-102

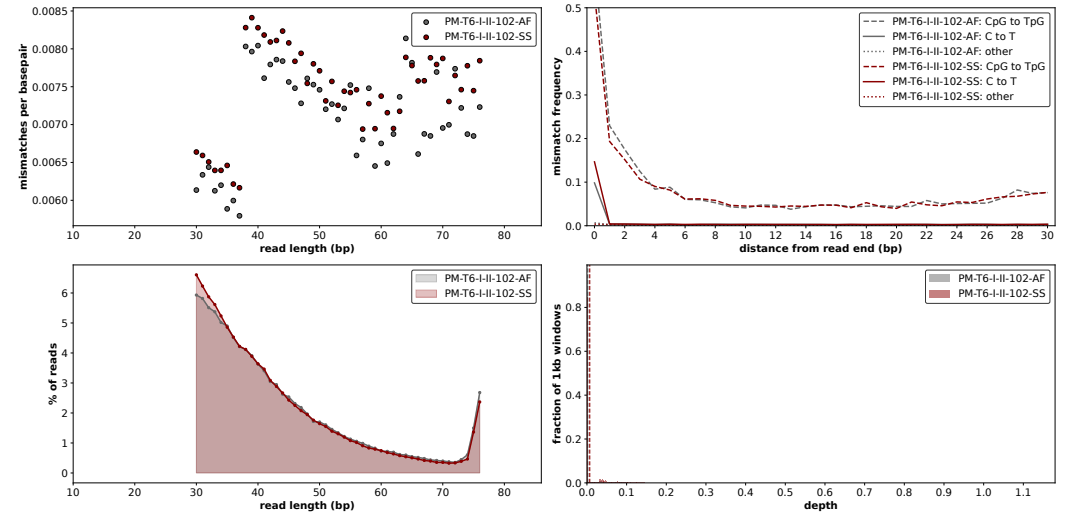

## I) PM-V6-I-II

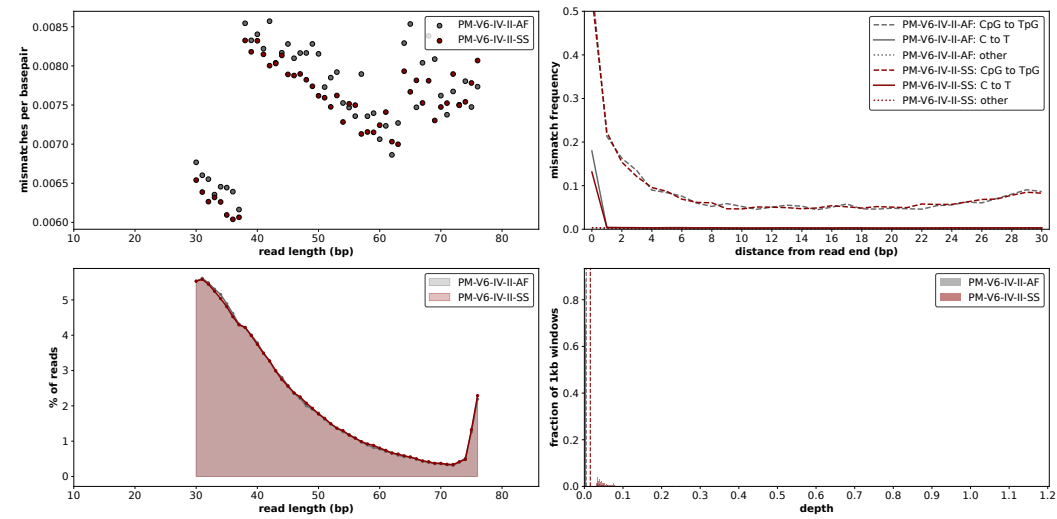

## J) PU-D2-VIII-101

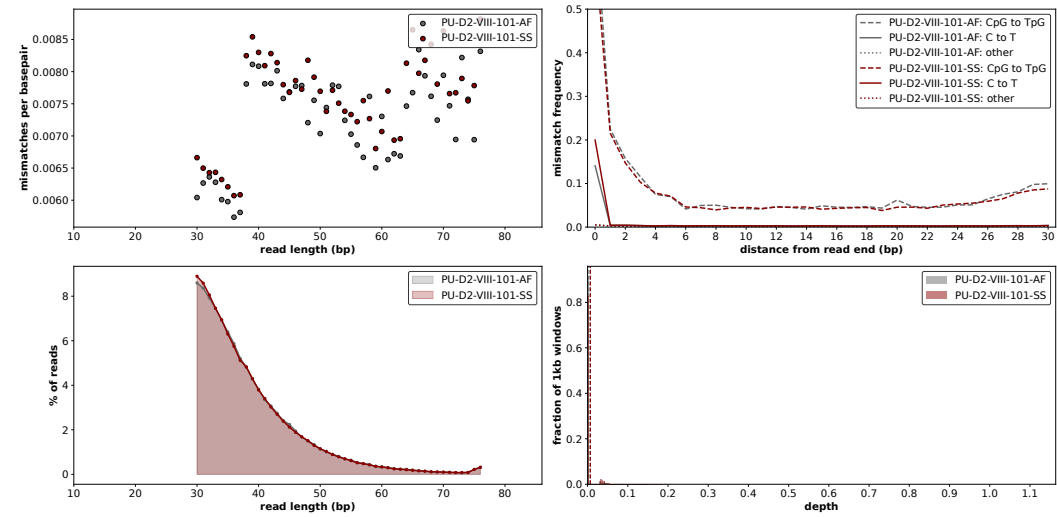

Supplement: S1 File — For each sample, 50 mg bone powder was sampled from the otic capsule of the petrous bone as described in Alberti et al. [7]. DNA was extracted using the methods of Dabney et al. [2] and quantified using a Qubit fluorometer high sensitivity assay of 1µl of the DNA extracted. The estimated concentration was multiplied by the 25 μl elution volume to obtain an estimate of the total DNA yield. Single stranded libraries [3] were prepared from the extracts and low level sequencing carried out on an Illumina NextSeq platform [25]. The resulting data were mapped to the reference genome of the polar bear using the procedure described in Barlow et al. [32,33] to provide an estimate of endogenous content. S2 Fig. Nucleotide misincorporation and read length plots for deeper sequencing of sample O34-16, for the standard single-stranded library protocol (SS; red) and the amplification-free protocol (AF; grey). Plots were generated using AMBER [29] with default parameters. (A) mismatch frequency and (B) nucleotide misincorporation at each position in the read, (C) read length and (D) depth in 1kb windows. Note that the amplification-free library was sequenced using a 75bp single-end sequencing method, and the single-stranded library using 75bp paired-end method. This leads to an accumulation of all fragments that were > 75bp at a read length of 74-75bp for the amplification-free library which can be seen in panel C (AF; grey). S3 Fig. Nucleotide misincorporation plots for all samples, for the standard single-stranded library protocol (SS) and the amplification-free protocol (AF). Plots were generated using AMBER [29] with default parameters. Panels for each figure are the same as defined in S2 Fig legend. S1 Table. Wetlab information. S2 Table. Sequence data statistics. S3 Table. Subsampled sequence data statistics. S4 Table. Published cave bear datasets included in population clustering analysis. S5 Table. Oligonucleotides used in this study. Lowercase bases indicate index sequenc [file pone.0319573.s001.zip › S3_Figure_AMBERlowcovs.pdf]
